# Cross-cultural adaptation and validation of the Hospital Survey on Patient Safety Culture 2.0 – Brazilian version

**DOI:** 10.1186/s12913-022-08890-7

**Published:** 2023-01-14

**Authors:** Claudia Tartaglia Reis, Josué Laguardia, Paola Bruno de Araújo Andreoli, Cassimiro Nogueira Júnior, Mônica Martins

**Affiliations:** 1 Ministério da Saúde, Planejamento e Gestão SMS Cataguases (MG), Rua Manoel Ramos Trindade 76/201, Cataguases, MG 36770-014 Brazil; 2 Instituto de Comunicação e Informação Científica e Tecnológica em Saúde, Fundação Oswaldo Cruz, Rio de Janeiro, RJ Brazil; 3 Quality, Patient Safety and Clinical Outcomes Manager – Hospital Alemão Oswaldo Cruz, São Paulo, SP Brazil; 4 Hospital Alemão Oswaldo Cruz, São Paulo, SP Brazil; 5 Escola Nacional de Saúde Pública Sérgio Arouca, Fundação Oswaldo Cruz, Rio de Janeiro, RJ Brazil

**Keywords:** Organizational culture, Safety culture measurement, Patient safety, Health care quality, Cross-cultural adaptation, Validity and reliability, Questionnaire, HSOPSC 2.0

## Abstract

**Background:**

Patient safety culture concerns the values, beliefs and standards shared by an organisation’s health staff and other personnel which influence their care provision actions and conduct. Several countries have made a priority of strengthening patient safety culture to improve the quality and safety of health care. In this direction, measuring the patient safety culture through validated instruments is a strategy applied worldwide. The purpose of this study was to adapt transculturally and validate the HSOPSC 2.0 to Brazilian Portuguese and the hospital context in Brazil.

**Methods:**

Of the various validated scales for measuring safety culture, the instrument most used internationally is the Hospital Survey on Patient Safety Culture (HSOPSC) developed by the United States Agency for Healthcare Research and Quality in 2004 and revised in 2019, when version 2.0 was released. Adaptation was conducted on a universalist approach and the adapted instrument was then applied to a sample of 2,702 respondents (56% response rate) comprising staff of a large general hospital in the city of São Paulo. Construct validity was investigated by Exploratory Structural Equation Modelling-within-Confirmatory Factor Analysis (ESEM-within-CFA) and reliability was measured in each dimension by means of Cronbach alpha coefficients.

**Results:**

ESEM fit indexes showed good data fit with the proposed model: χ2 = 634.425 df = 221 χ2/df ratio = 2.9 *p*-value < 0.0000; RMSEA = 0.045 (90% C.I. = 0.041—0.050) and probability RMSEA <  = .05 = 0.963; CFI = 0.986; TLI = 0.968. However, ten items had loads lower than 0.4. Cronbach alpha values were 0.6 or more for all dimensions, except Handoffs and information exchange ($\alpha$= 0.50) and Staffing and work pace ($\alpha$ = 0.41).

**Conclusion:**

The psychometric properties of the Brazilian version were found to be satisfactory, demonstrating good internal consistency and construct validity as expressed by estimates of reliability and indexes of model fit. However, given factor loadings smaller than 0.4 observed in ten items and considering that the scale translated and adapted to Portuguese was tested on a single sample during the Covid-19 pandemic, the authors recognize the need for it to be tested on other samples in Brazil to investigate its validity.

## Background

Over the past twenty years, the patient safety movement has developed in several directions. At first, studies endeavoured to understand the magnitude of the problems related to safety incidents, the factors contributing to such occurrences and their consequences and harm to patients, known as adverse events. The focus later broadened to take in human and organisational considerations that might contribute to preventing the occurrence of these undesirable outcomes for patients by measures to improve the health service context [1–3]. In that regard, several countries made a priority of strengthening patient safety culture with a view to improving the quality and safety of health care. Recently, the subject has gained greater prominence as countries face concerns relating to the Covid-19 pandemic [4, 5].

Increasingly, the discussion has addressed the roles of leadership, communication, teamwork, and other dimensions of the safety culture shared among health organisation staffs [6]. The current agenda directed to improving the quality of patient care includes topics related to building a safety culture that is open and fair to both health personnel and patients, encourages learning and prioritises open communication among work teams. The World Health Organisation also stresses the importance of leadership support for building a robust safety culture and assuring psychological security in the workplace, avoiding overwork of health staffs, and encouraging the reporting and investigation of safety incidents to benefit continued learning [7].

Patient safety culture, an integral part of organisational culture, concerns the values, beliefs and standards that are shared by an organisation’s health personnel and other workers and influence their manner, actions and conduct in relation to safety [8]. In that light, measures directed to strengthening safety culture have become a subject for ample discussion among health organisation managers and researchers all over the world. To assist them in planning investments in improvement, it has been recommended to measure patient safety culture using validated instruments, a strategy applied worldwide [9].

Of the various validated instruments for measuring safety culture, the Hospital Survey on Patient Safety Culture – Version 1.0 (HSOPSC Version 1.0), developed by the United States Agency for Healthcare Research and Quality (AHRQ) in 2004, is the instrument most used internationally [9, 10]. A version adapted transculturally to Brazilian Portuguese and the hospital context in Brazil was the subject of a previous study [11] and, with AHRQ authorisation to be used freely, is being applied widely in Brazil [12–15].

Over the years, United States, and international users of the HSOPSC 1.0 sent the AHRQ reports and suggestions for change in the instrument. These included notably: i) reviewing the wording of complex and hard-to-translate items; ii) including the response option “Does not apply or Don’t know”; iii) altering the structure of the dimension evaluating response to errors, to contemplate the concept of a culture of fairness; iv) the number of negatively worded items was reduced; and v) revising items relating to hospital job positions and work areas [16]. The instrument then underwent review and several modifications and, in December 2019, the AHRQ released a new version, the HSOPSC 2.0 [8].

In Brazil, building a strong safety culture in health organisations is the core aim of the National Patient Safety Programme [17] and measuring safety culture in hospitals has been encouraged as a first step in this process and essential to evaluating changes occurring over time. In that connection, a measurement instrument that is current, valid, and reliable is extremely valuable, which motivated us to carry out this study.

## Methods

### Study aim

The purpose of this study was to adapt transculturally and validate the HSOPSC 2.0 to Brazilian Portuguese and the hospital context in Brazil.

### Study design

The study, with AHRQ authorisation, was conducted in two stages. In the first, the universalist approach was applied to evaluating equivalences in semantics, concepts, and questionnaire items [18, 19]. Here, the instrument was carefully translated and evaluated by a panel of experts. A pre-test was then carried out to assess the operational equivalence, verbal comprehension, and clarity of the items. The second stage was a cross-sectional observation study to evaluate equivalence in measurement by means of psychometric analyses of the instrument’s reliability and validity.

### Instrument

The HSOPSC 2.0 comprises 40 items (Table 1), 32 of which are grouped into 10 dimensions which make up the concept of “patient safety culture”. These 32 items are measured on a five-point Likert scale for agreement (from “Strongly disagree” to “Strongly agree”) or frequency (“Never” to “Always”), plus a response option “Does not apply or Don’t know” (Table 1). The instrument also included two single-item outcome measurements, one relating to how often incidents were reported and the other asking respondents to give a patient safety rating in their unit/work area. The six remaining questions request participant data on job position, work unit, length of time working at the hospital and in the present unit/area, weekly hours worked and whether in direct contact with patients.

**Table 1:** Safety Culture Dimensions and component items listed in order of appearance in U.S. HSOPSC version 2.0

Safety Culture Dimension	Items (N)	**Item**
**Teamwork**	3	A1. In this unit, we work together as an effective team
		A8. During busy times, staff in this unit help each other
		A9R. There is a problem with disrespectful behavior by those working in this unit
**Staffing and Work Pace**	4	A2R. In this unit, we have enough staff to handle the workload
		A3. Staff in this unit work longer hours than is best for patient care
		A5R. This unit relies too much on temporary, float, or PRN staff
		A11R. The work pace in this unit is so rushed that it negatively affects patient safety
**Organizational Learning -Continuous Improvement**	3	A4. This unit regularly reviews work processes to determine if changes are needed to improve patient safety
		A12. In this unit, changes to improve patient safety are evaluated to see how well they worked
		A14R. This unit lets the same patient safety problems keep happening
**Response to Error**	4	A6R. In this unit, staff feel like their mistakes are held against them
		A7R. When an event is reported in this unit, it feels like the person is being written up, not the problem
		A10. When staff make errors, this unit focuses on learning rather than blaming individuals
		A13R. In this unit, there is a lack of support for staff involved in patient safety errors
**Supervisor, Manager, or Clinical Leader Support for Patient Safety**	3	B1. My supervisor, manager, or clinical leader seriously considers staff suggestions for improving patient safety
		B2R. My supervisor, manager, or clinical leader wants us to work faster during busy times, even if it means taking shortcuts
		B3. My supervisor, manager, or clinical leader takes action to address patient safety concerns that are brought to their attention
**Communication About Error**	3	C1. We are informed about errors that happen in this unit
		C2. When errors happen in this unit, we discuss ways to prevent them from happening again
		C3. In this unit, we are informed about changes that are made based on event reports
**Communication Openness**	4	C4. In this unit, staff speak up if they see something that may negatively affect patient care
		C5. When staff in this unit see someone with more authority doing something unsafe for patients, they speak up
		C6. When staff in this unit speak up, those with more authority are open to their patient safety concerns
		C7R. In this unit, staff are afraid to ask questions when something does not seem right
**Reporting Patient Safety Events**	2	D1. When a mistake is caught and corrected before reaching the patient, how often is this reported?
		D2. When a mistake reaches the patient and could have harmed the patient, but did not, how often is this reported?
**Hospital Management Support for Patient Safety**	3	F1. The actions of hospital management show that patient safety is a top priority
		F2. Hospital management provides adequate resources to improve patient safety
		F3R. Hospital management seems interested in patient safety only after an adverse event happens
**Handoffs and Information Exchange**	3	F4R. When transferring patients from one unit to another, important information is often left out
		F5R. During shift changes, important patient care information is often left out
		F6. During shift changes, there is adequate time to exchange all key patient care information

*R*- reverse items

### Evaluation of conceptual, semantic and item equivalences of the HSOPSC 2.0 – Brazilian version

The U.S. HSOPSC 2.0 version was rendered into Portuguese by two translators, to generate two translations (T1 and T2). The translators are all professionals and native speakers of English. Three researchers then compared the translated versions, T1 and T2, with the original in English, examining for conceptual and semantic equivalence. After decisions by consensus arbitrated by the research team, a synthesis of the translations, T12, was then produced. The bilingual research team that produced the T12 synthesis comprised a public health nurse working in patient safety culture assessment and epidemiology; a researcher into health service assessment, care quality and patient safety; and a doctor specialising in epidemiology, health information research and psychometric assessment of measurement scales. The T12 synthesis version was then backtranslated into English by two translators working independently to produce versions R1 and R2.

Semantic, conceptual, experiential and item equivalences were evaluated by a panel of experts based on the original version in English, plus versions T1, T2, T12, R1 and R2. The expert panel comprised 19 professionals representing the fields of nursing, medicine, physiotherapy, administration, risk and quality management and hospital patient safety unit management. Panel participants also included undergraduate and postgraduate researchers and professors in the fields of collective health, quality of care and patient safety, medicine, and nursing. The participants received an invitation by email and, after acceptance, a form containing all the items of the instrument in the six versions described above. For each item, participants were asked to choose one response option on a scale of 4, ranging from “Item is not representative” to “Item is representative”, as regards semantic, conceptual, experiential and item equivalence. Where participants did not choose the “Item is representative” option, they were asked, in an open-ended question, to comment on the item and suggest possible adjustments to its wording. The questionnaire containing all the translated versions was developed using Research Electronic Data Capture (REDCap https://redcapbrasil.com.br) and forwarded to expert panel participants by e-mail using an individual personalised link for each expert. Their responses were stored on the REDCap platform and exported to Excel® for analysis.

The version obtained from the expert panel contributions underwent pre-testing in a convenience sample of 29 hospital staff members, including doctors, nurses, pharmacists, physiotherapists, nutritionists, administrators, and nursing technicians, to assess operational equivalence, verbal comprehension, and item clarity. The participating professionals performed care and managerial functions at the hospital. Participants were asked to indicate how well they understood each item of the instrument on a Likert scale ranging from “I did not understand at all” to “I understood perfectly and had no doubts”. After analysis of the pre-test results, the final version was produced for use in the cross-sectional study to evaluate its psychometric properties.

### Cross-sectional study

#### Setting, sample and data collection

The study sample comprised all the staff of a large general hospital, open to outside practitioners and with international accreditation, in the city of São Paulo (Brazil). All staff present at the hospital during data collection were included, except those belonging to partner firms providing laboratory, blood bank, anatomical pathology, and security services at the hospital. This exclusion criterion responded to the high staff turnover in these services and the impossibility of assuring that they perceived the organisational culture shared in the work environment. Staff were invited to participate in the study by means of an invitation posted in usual institutional communication channels. The strategy for encouraging staff to join the study was to persuade the institution’s leaders to notify their teams directly. During data collection, leaders were provided with weekly reports on percentage response, so that local campaigns could be reinforced to boost involvement and response to the instrument.

In view of the Covid-19 pandemic, the data collection strategy took the form of an online survey via a form compiled in REDCap and using the HSOPSC version 2.0, translated and adapted based on the pre-test results. The survey was published on the hospital intranet and a link was provided for data collection via the web.

After reading and agreeing to the Informed Consent Form (ICF), participants gained access to complete the questionnaire online. In all, 3,699 staff members were invited to participate. Data were collected from 17 August to 18 September 2021. To preserve participant anonymity, no information was collected on age, gender, or schooling.

#### Data analysis

Descriptive statistics were estimated to characterise participants by job position, hospital work unit, length of employment at the hospital, hours worked per week and type of contact with patients.

Mean scores and percentage positive responses were calculated for each dimension. All negatively worded items were reverse coded. Positive responses were taken to be those to which respondents marked the options “Strongly agree” or “Agree”, or “Always” or “Most of the time” for positively worded items and “Strongly disagree” or “Disagree”, or “Never” or “Rarely” for negatively worded items.

Multivariate normality was evaluated using the Small, Srivastrava and Mardia tests of multivariate skewness and kurtosis, as well a Small-based omnibus test [20] using the “ + 2/-2” guidelines. Construct reliability was ascertained using Cronbach alpha estimates for the extent to which results obtained from the Brazilian version of the HSOPSC 2.0 could be replicated and compared with other instrument validation studies [16, 10, 21]. Reliability was assessed by way of internal consistency. Cronbach alpha coefficients were estimated for each dimension, using SPSS 26.0, and following the dimensional structure of the original model. Values above 0.6 were considered acceptable [22].

Construct validity was investigated by way of Exploratory Structural Equation Modelling (ESEM)-within-CFA, which assumes that the resulting measurement structure of an ESEM factor model will remain stable when transformed into a CFA model [23]. Although considered basically a confirmatory technique [24], ESEM was chosen for it combining the findings of the confirmatory and exploratory factor analysis (CFA and EFA), making it possible to explore the underlying factor structure without the constraints imposed by CFA, as well as estimating the CFA parameters of tests of model fit [25].

In ESEM, model fit was evaluated by χ2, p-value and three indices: root mean square error of approximation (RMSEA) with 90% confidence intervals (CI), comparative fit index (CFI) and Tucker-Lewis’s index (TLI). CFI and TLI are incremental measures of fit, enabling the proposed model to be compared with an independence (null) model. The values of these indices range from 0 to 1, where values greater than 0.90 indicate proper model fit. Absolute model fit was evaluated by RMSEA, which estimates how well the parameters reproduce the population variance. It is a parsimonious correction index, in that it contains a penalty for the number of parameters estimated, expressed in degrees of freedom. The smaller the RMSEA value, the better the fit. Values close to or smaller than 0.06 indicate reasonable model fit, while values greater than 0.10 indicate poor fit and that the model should be rejected [26]. Discriminant validity was determined by examining the correlations among the dimensions: a value greater than 0.70 was considered too high and thus inappropriate [27]. Factor loading on each item of the instrument was evaluated, considering loading 0.4 as appropriate, and item uniqueness was established by way of residual error variances (> 0.10, but < 0.90) [23]. In the ESEM models, cross-loadings were targeted to be as close to zero as possible to reflect the confirmatory approach of ESEM.

ESEM was performed using the MPlus Version 8.1 Base Programme and the Mixture Add-On Single-User licence N. SCBMX80001318.

## Results

The panel of experts suggested alterations to the synthesis version to be subjected to pre-testing. It was suggested that the first question, on the participant’s job position, include certain professional categories and exclude others to match the Brazilian context. The word "staff" that is part of questions A2, A3r, A5r, A6r, A8, A10, A13r, B1, C4, C5, C6 and C7r was translated into Portuguese as the equivalent of “professionals”. Minor adjustments were also made to the wording another items. Item A9r, the sentence “there is a problem with disrespectful behaviour” was translated into Portuguese as the equivalent of “there are problems with disrespectful behaviour”. In the three questions in section B, “my supervisor, manager, or clinical leader” was translated to “my supervisor, manager or chief of staff/clinic”. In items C5 and C6, “speak up” was translated into Portuguese as the equivalent of “speak openly”.

Most items of the instrument were satisfactorily understood in the pre-test, but 3 items (A3r, A5r, A14r) called for special attention. After the team of researchers conducting the study had considered the participants’ doubts and suggestions and arrived at a consensus, minor adjustments were made to the wording of these items. The A3r item “Staff in this unit work longer hours than is best for patient care” was translated into Portuguese as the equivalent of “Professionals in this unit work longer hours than would be desirable to provide the best patient care”. Due to issues related to understanding and the different types of employment contracts in the Brazilian hospital context, the A5r item "This unit relies too much on temporary, float, or PRN staff" was adapted to "This unit relies too much on personnel engaged under third-party contracts or any other kind of temporary contract”. In item A14, "keep happening" was adapted to “continue to occur”. The final version resulting from this step was used in the cross-sectional study.

### Cross-sectional study

#### Study sample, descriptive statistics and reliability

Of the 3,669 staff invited to take part, 2,121 responded to the instrument. After evaluation for data completion quality, 25 instruments were excluded for non-completion of at least 50% of items and 24 were excluded because participants selected the same option in items worded positively and negatively regarding patient safety, indicating a lack of the attention necessary to respond to the questionnaire [8]. Accordingly, 2,072 instruments were considered appropriate for analysis, giving a 56% response rate.

Study participant characteristics (Table 2) indicated a range of professional categories, predominantly nursing-related (39.5%), plus doctors (7.4%), pharmacists, pharmacy technicians and auxiliaries (5.4%). Participants also included personnel of support services (8.6%), such as catering, infrastructure, nutrition and dietetics and cleaning. Approximately 70% of participants had been working at the hospital for more than a year and 71% had worked in their present area or sector of the hospital for more than one year. Most of the participants (90.5%) worked more than 30 h a week and 68.5% were in direct interaction or contact with patients.

**Table 2:** Characteristics of Study Participants (*N* = 2,072)

Characteristics	(N)	%
Job/Function		
Nurse	349	16.8
Nursing Technician	438	21.1
Nursing Auxiliary	6	.3
Nursing Intern	24	1.2
Doctor	113	5.5
Hospitalist	40	1.9
Medical Resident	1	.0
Nutritionist	33	1.6
Pharmacist, Pharmacy Technician or Auxiliary	111	5.4
Physiotherapist, Occupational Therapist or Speech Therapist	107	5.2
Psychologist	3	.1
Technician (Laboratory, X-ray, Electrocardiogram)	15	.7
Supervisor, Department Manager, Head of Team/Clinic	70	3.4
Superintendent, Director General, CEO	9	.4
Catering, Maintenance, or Infrastructure	40	1.9
Nutrition and Dietetics	93	4.5
Cleaning Service	46	2.2
Information Technology, Health Information Services and Medical Computation	51	2.5
Transport	8	.4
Unit Personnel or Secretary, Receptionist, Administration	251	12.1
Other	264	12.7
Time working at the hospital		
Less than 1 year	634	30.6
1 to 5 years	863	41.7
6 to 10 years	296	14.3
11 years or more	279	13.5
Time working in present unit/work area		
Less than 1 year	767	37.0
1 to 5 years	903	43.6
6 to 10 years	234	11.3
11 years or more	168	8.1
Hours worked per week		
Less than 30 h per week	192	9.3
30 to 40 h per week	1062	51.3
More than 40 h per week	813	39.2
Not known	5	.2
Direct Interaction/Contact with Patients		
Yes	1419	68.5
No	652	31.5
Not known	1	.0

Table 3 juxtaposes the reliability and the percentage positive responses to safety culture dimensions of the Brazilian version of the HSOPSC 2.0 with the results obtained in the first three studies published [28, 10, 21]. The percentage positive responses obtained in this study for the dimensions of patient safety culture ranged from 51 to 82%. The highest percentage positive responses were given in the following dimensions: “Supervisor, manager, or clinical support for patient safety” (82%), “Teamwork” (81%), “Reporting patient safety event” (77%), “Organisational learning – Continuous improvement” (76%) and “Hospital management support for patient safety” (76%). Of all the dimensions, “Staffing and work pace” obtained the lowest percentage (54%) positive responses.

**Table 3:** Reliability and percentage positive responses to safety culture dimensions of the HSOPSC 2.0 Brazilian version

Safety culture dimensions (composite measure items)	Cronbach’s $\boldsymbol{\alpha }$				Percentage Positive Responses		
	**This study** **(*N* = 2,072)**	Original HSOPSC 2.0 [28] (*N* = 4,345)	Korean-HSOPSC 2.0 [10] (*N* = 526)	Indonesian HSOPSC 2.0 [21] (*N* = 220)	**This study**	Original HSOPSC 2.0 [28]	Korean-HSOPSC 2.0 [10]
**Teamwork (A1, A8, A9R)**	**.68**	.76	.77	.76	**81**	81	62
**Staffing and Work Pace (A2, A3R, A5R, A11R)**	**.47**	.67	.61	.73	**54**	56	13
**Organizational learning- Continuous improvement (A4, A12, A14R)**	**.60**	.76	.71	.76	**76**	72	54
**Response to error (A6R, A7R, A10, A13R)**	**.76**	.83	.72	.68	**62**	61	22
**Supervisor, Manager, or Clinical Leader Support for Patient Safety (B1, B2R, B3)**	**.71**	.77	.75	.74	**82**	81	69
**Communication about error (C1, C2, C3)**	**.87**	.89	.83	.73	**70**	68	50
**Communication Openness (C4, C5, C6, C7R)**	**.76**	.83	.73	.67	**67**	76	38
**Reporting Patient Safety Event (D1, D2)**	**.81**	.75	.73	.81	**77**	74	40
**Hospital Management Support for Patient Safety (F1, F2, F3R)**	**.62**	.77	.72	.75	**76**	68	31
**Handoffs and Information Exchange (F4R, F5R, F6)**	**.50**	.72	.72	.76	**61**	58	63

Results of Small, Srivastava and Mardia tests of multivariate skew and kurtosis, as well as an omnibus test of multivariate normality based on Small's test, returned *p*-values of less than 0.05, rejecting the null hypothesis of multivariate normality. The dataset cannot be assumed to follow a multivariate normal distribution.

Cronbach alpha values (Table 3) were 0.6 or more for all dimensions, except Handoffs and information exchange ($\alpha$= 0.50) and Staffing and work pace ($\alpha$ = 0.41).

#### Exploratory Structural Equation Modelling (ESEM)-within-CFA

ESEM returned fit indices showing good fit between the data and the proposed model. They were as follows: χ2 = 634.425 df = 221 χ2/df ratio = 2.9 *P*-Value = 0.0000; RMSEA = 0.045 (90% C.I. = 0.041—0.050) Probability RMSEA <  = 0.05 = 0.963; CFI = 0.986; TLI = 0.968.

The correlations among the 10 dimensions (Table 4) ranged from 0.034 (between “Teamwork” and “Staffing and work pace”) to 0.634 (between “Communication about errors” and “Reporting safety patient events”). “Teamwork” showed little correlation (0.034) with “Staffing and work pace”; correlation between “Staffing and work pace” and “Communication openness”, “Reporting patient safety events” and “Handoffs and information exchange” was weak: 0.069, 0.041 and 0.079, respectively. No excessively strong correlations were observed.

**Table 4:** Correlation matrix among ten dimensions of the Hospital Survey on patient safety culture 2.0, Brazil, 2022

Dimension	1	2	3	4	5	6	7	8	9
2	0.034^a^								
3	0.175	0.201							
4	0.137	0.121	0.297						
5	0.550	0.145	0.281	0.139					
6	0.296	0.118	0.200	0.130	0.537				
7	0.320	0.069^a^	0.179	0.116	0.482	0.621			
8	0.217	0.041^a^	0.177	0.075	0.421	0.634	0.568		
9	0.360	0.103	0.211	0.197	0.593	0.522	0.445	0.504	
10	0.133	0.079^a^	0.338	0.143	0.369	0.285	0.226	0.288	0.323

All correlations were below *r*^*2*^ = 0.7. Correlation between 1 and 2, 2 and 7, 2 and 8, and 2 and 10 are not significant (*P* > 0.05). The remaining correlations are significant at *P* < *0.05*

1. Teamwork

2. Staffing and Work Pace

3. Organizational Learning—Continuous Improvement

4. Response to Error

5. Supervisor, Manager, or Clinical Leader Support for Patient Safety

6. Communication About Error

7. Communication Openness

8. Reporting Patient Safety Events

9. Hospital Management Support for Patient Safety

10. Handoffs and Information Exchange

^a^Not significant

The factor loadings obtained in ESEM (Table 5) were compared to the dimensional structure of the original model (Table 1). All items comprising the dimensions “Supervisor, Manager, or Clinical Leader Support for patient safety”, “Communication about error” and “Reporting Patient Safety event” had factor loadings greater than 0.4. The dimensions “Communication Openness”, “Hospital Management Support for patient safety”, “Handoffs and information exchange”, “Teamwork”, “Staffing and workpace” and “Response to error” had at least 1 item with a factor loading lower than 0.4. And in the “Organizational learning-continuous improvement” dimension, only one item had a factor loading greater than 0.4.

**Table 5:** Factor Loadings, overall *R*-Square and residual variance of the Brazilian HSOPSC version 2.0 items estimated by Exploratory Structural Equation Modelling

Safety culture dimension (Component items)	Factor Loadings	Standard Error	*R*-Square Estimate*	Residual Variance
Teamwork				
A1	0.609	0.000	0.697	0.303
A8	0.593	0.000	0.706	0.294
A9R	0.243	0.000	0.448	0.552
Staffing and Work Pace				
A2	0.444	0.090	0.648	0.352
A3R	0.508	0.000	0.368	0.632
A5R	0.271	0.000	0.214	0.786
A11R	0.344	0.000	0.686	0.314
Organizational learning – Continuous improvement				
A4	0.031	0.447	0.585	0.414
A12	0.045	0.316	0.598	0.402
A14R	0.602	0.000	0.735	0.265
Response to error				
A6R	0.548	0.000	0.736	0.264
A7R	0.406	0.000	0.668	0.332
A10	0.275	0.000	0.574	0.426
A13R	0.125	0.000	0.704	0.296
Supervisor, Manager, or Clinical Leader Support for Patient Safety				
B1	0.876	0.000	0.757	0.243
B2R	0.577	0.000	0.529	0.471
B3	0.725	0.000	0.693	0.307
Communication about error				
C1	0.790	0.000	0.727	0.273
C2	0.764	0.000	0.855	0.145
C3	0.673	0.000	0.763	0.237
Communication Openness				
C4	0.536	0.000	0.712	0.288
C5	0.670	0.000	0.654	0.346
C6	0.619	0.000	0.731	0.269
C7R	0.175	0.001	0.462	0.538
Reporting Patient Safety Event				
D1	0.891	0.000	0.789	0.211
D2	0.959	0.000	0.791	0.209
Hospital Management Support for Patient Safety				
F1	0.837	0.000	0.812	0.188
F2	0.871	0.000	0.859	0.141
F3R	0.153	0.001	0.361	0.639
Handoffs and Information Exchange				
F4R	0.818	0.000	0.736	0.264
F5R	0.748	0.000	0.611	0.389
F6	0.088	0.013	0.371	0.629

*p*-value = 0.000

## Discussion

Following the methodological process for translating and evaluating equivalences in semantics, concepts, and items, which included an expert panel and pre-testing with hospital staffs, all the items of the original model were found to be relevant and applicable to the Brazilian hospital context, even though it is highly heterogeneous. Public and private hospitals in Brazil display different degrees of development and maturity as regards care quality and patient safety. Accordingly, similarly to previous studies of transcultural adaptation of the HSOPSC version 1.0 to the Brazilian context [11, 29, 30], all the items contained in the original version of the instrument were retained in the final version produced in the study reported here, as in the Indonesian study [21]. This study thus differed from the Korean study [10], which excluded the item A5R “This unit relies too much on temporary, float, or PRN staff”. At the transcultural adaptation stage, this item was considered relevant to the Brazilian context, but it was adjusted semantically to the cultural context in which hospital staff are engaged in Brazil. In the final version used in the cross-sectional study, it read “This unit relies too much on personnel engaged under third-party contracts or any other kind of temporary contract”.

The 56% response rate obtained in this study was higher than those of Brazilian studies of transcultural adaptation of the HSOPSC version 1.0. A study at two large hospitals in southeast Brazil obtained a response rate of 38.5% [11]; another, at a large hospital in a major metropolis in Brazil had a response rate of 18.7% [30]. It is essential to achieve a high response rate to assure robust psychometric analyses and, in parallel, to understand the capillarity of safety culture in the context where the instrument is applied.

Although the population of this study included a variety of professional categories (unlike the Korean [10] and Indonesian [21] studies to evaluate the HSOPSC 2.0, which included exclusively nurses), nursing-related categories did account for the largest percentage (39.5%) of participants. Similarly, the U.S. Study (40%) [28] and a systematic review on use of the HSOPSC version 1.0 [9] found nursing personnel to be the most numerous participants in studies to evaluate safety culture in hospitals.

This study was not designed primarily to measure patient safety culture, although the information on safety culture in the participating hospital did reveal five dimensions to be strong (≥ 75% positive responses): “Supervisor, manager, or clinical support for patient safety” (82%), “Teamwork” (81%), “Reporting patient safety event” (77%), “Organisational learning – Continuous improvement” (76%) and “Hospital management support for patient safety” (76%). No dimension was found to be weak (≤ 50% positive responses). However, the lowest percentages were scored by the dimensions “Staffing and work pace” (54%), “Handoffs and information exchange” (61%) and “Response to error” (62%). The percentage positive responses returned in this study in patient safety culture dimensions were close to those found in the original U.S. Study [28] and were higher than perceptions of safety culture reported by nurses in the Korean study [10].

The lowest percentage (54%) of positive responses scored in the “Staffing and work pace” dimension illustrates the extent to which working conditions (human resources and workload) are essential to assuring quality health care and patient safety. This dimension was found to be weak in around 60% of studies that have evaluated safety culture using the HSOPSC [9], suggesting that personnel feel overloaded for staffing-related reasons. This scenario became especially acute during the Covid-19 pandemic. The period early in the pandemic (which was when data were collected for this study) was characterised by high demand for admission of severely ill patients and absences of front-line personnel from illness. Similarly, it was this dimension that scored the lowest percentage of positive responses (56%) in the U.S. study [28] and (13%) in the Korean study [10].

“Handoffs and information exchange” was the dimension that returned the second largest percentage positive responses (61%), similarly to the U.S. Study, where it obtained 58% [28]. This dimension, which has to do with information about patient care that is important when transfers between units and shift handovers take place [8], was found to be weak in 30% (*n* = 10) of the studies that used the HSOPSC 1.0 [9]. It is a communication-related dimension of major importance in the safety culture shared among hospital staffs, because when strong it can reduce the risk of avoidable incidents and fragmentation of patient care resulting from loss of important information about treatment [31, 32].

“Response to error” was the dimension with the third lowest percentage positive responses (62%), similarly to the U.S. study [28]. In the original HSOPSC version 1.0 model, this dimension was termed “Nonpunitive response to error”. In the original HSOPSC 2.0 model, where this dimension had previously rested on a non-punitive approach to staff in the event of error. The updated version (2.0) of this dimension, now titled “Response to error”, in addition to contemplating a culture of not blaming staff, came to incorporate concepts of a culture of fairness comprising both accountability and learning from occurrences of error. The culture of fairness recommends treating staff fairly in the event of errors and focusing on learning from mistakes and supporting the team involved in the safety incident [8]. Although “Response to error” was not classified here as a weak, the participating hospital would benefit from investments and initiatives to improve this dimension.

Overall, the findings from examining the psychometric properties of the Brazilian version of the HSOPSC 2.0 were positive, displaying good internal consistency and construct validity, as expressed in the reliability estimates and model fit indices. However, the low factor loadings present in 10 items would entail removing these items to explore a factor model that best fits the data collected in the sample of this study. For several reasons, we chose not to go this way. The theoretical basis underlying the AHRQ SOPS survey is recognised as applicable to the reality of Brazilian health services [29, 30]. Brazil's National Patient Safety Programme highlights the importance of building a strengthened patient safety culture and encourages its measurement to monitor advances and weaknesses in this area. Furthermore, the programme encourages sharing a fair culture of reporting, accountability and learning in health services. Also, all stages of the translation and cross-cultural adaptation were carried out with methodological rigor, which included a scrutiny of translation possibilities and alternatives, and was supported by an expert panel. Lastly, data collection for validation of version 2.0 took place in a single hospital during the Covid-19 pandemic period, which may have affected the fit of the data to the ten-factor model.

Unlike the studies in Korea [10], Indonesia [21], and the USA [28], which returned Cronbach alpha values more than 0.60 in the ten dimensions of safety culture (Table 3), here, satisfactory results were obtained in 8 of the 10 dimensions. Cronbach alphas for the “Staffing and work pace” and “Handoffs and information exchange” dimensions were 0.47 and 0.50, respectively. However, these results were better than those of the validity and reliability study of version 1.0 for Brazil [30], which returned low Cronbach alpha values for “Overall perceptions of patient safety” (0.41), “Teamwork across units” (0.45), “Staffing” (0.46) and “Non-punitive response to error” (0.54).

### Limitations

Some limitations of this study must be described. The study sample called on the participation of staff members of a single hospital with a history of accreditation in a large metropolis in Brazil, which limited the scope for generalising the safety culture findings to Brazilian hospitals. Brazil’s hospital system is heterogeneous and uneven in its maturity as regards strategies to assure care quality and patient safety.

Not only was the Brazilian version of the HSOPSC 2.0 tested in only one hospital, but there are also not many other published studies available for the purpose of comparing its psychometric properties. Relying on the methodological rigour of the stages of translation and transcultural adaptation and for the reasons outlined above, it was decided here to retain the dimensional structure of the instrument as in the original model and it is stressed that this should be tested in other samples from hospitals in Brazil with different structural characteristics and accreditation status.

### Conclusion

This study resulted in a version of the HSOPSC 2.0 adapted to Portuguese, for the purpose of measuring patient safety culture in Brazilian hospitals. We encourage that the adapted instrument be tested on other samples in Brazil, to investigate its validity.

## Data Availability

The datasets used and/or analysed during the current study are available from the corresponding author on reasonable request.
